# Cytosolic and Nucleosolic Calcium-Regulated Molecular Networks in Response to Long-Term Treatment with Abscisic Acid and Methyl Jasmonate in *Arabidopsis thaliana*

**DOI:** 10.3390/genes13030524

**Published:** 2022-03-16

**Authors:** Doudou Wang, Feifei Huang, Pengcheng Yan, Yanli Nie, Lvli Chen, Jin Luo, Heping Zhao, Yingdian Wang, Shengcheng Han

**Affiliations:** 1 Beijing Key Laboratory of Gene Resource and Molecular Development, College of Life Sciences, Beijing Normal University, Beijing 100875, China; 202031200007@mail.bnu.edu.cn (D.W.); 201531200013@mail.bnu.edu.cn (F.H.); 201031200041@mail.bnu.edu.cn (Y.N.); 201721200006@mail.bnu.edu.cn (L.C.); 201621200041@mail.bnu.edu.cn (J.L.); hpzhao@bnu.edu.cn (H.Z.); ydwang@bnu.edu.cn (Y.W.); 2 Department of Computational Biology, Beijing Computing Center, Beijing 100094, China; yanpc@bcc.ac.cn

**Keywords:** cytosolic calcium-regulated hormone-responsive gene, nucleosolic calcium-regulated hormone-responsive gene, gene co-expression network, hub gene, *A. thaliana*

## Abstract

Calcium acts as a universal secondary messenger that transfers developmental cues and stress signals for gene expression and adaptive growth. A prior study showed that abiotic stresses induce mutually independent cytosolic Ca^2+^ ([Ca^2+^]_cyt_) and nucleosolic Ca^2+^ ([Ca^2+^]_nuc_) increases in *Arabidopsis thaliana* root cells. However, gene expression networks deciphering [Ca^2+^]_cyt_ and [Ca^2+^]_nuc_ signalling pathways remain elusive. Here, using transgenic *A. thaliana* to selectively impair abscisic acid (ABA)- or methyl jasmonate (MeJA)-induced [Ca^2+^]_cyt_ and [Ca^2+^]_nuc_ increases, we identified [Ca^2+^]_cyt_- and [Ca^2+^]_nuc_-regulated ABA- or MeJA-responsive genes with a genome oligo-array. Gene co-expression network analysis revealed four Ca^2+^ signal-decoding genes, *CAM1*, *CIPK8*, *GAD1*, *and CPN20*, as hub genes co-expressed with Ca^2+^-regulated hormone-responsive genes and hormone signalling genes. Luciferase complementation imaging assays showed interactions among CAM1, CIPK8, and GAD1; they also showed interactions with several proteins encoded by Ca^2+^-regulated hormone-responsive genes. Furthermore, CAM1 and CIPK8 were required for MeJA-induced stomatal closure; they were associated with ABA-inhibited seed germination. Quantitative reverse transcription polymerase chain reaction analysis showed the unique expression pattern of [Ca^2+^]-regulated hormone-responsive genes in *cam1*, *cipk8*, and *gad1*. This comprehensive understanding of distinct Ca^2+^ and hormonal signalling will allow the application of approaches to uncover novel molecular foundations for responses to developmental and stress signals in plants.

## 1. Introduction

Sessile plants respond to developmental and environmental cues through various secondary messengers and phytohormones. Calcium ion (Ca^2+^) is a universal secondary messenger involved in fertilisation, growth, and development; it responds to both biotic and abiotic stresses in plants [1,2,3]. In addition, plants produce hormones (e.g., abscisic acid (ABA) and jasmonic acid (JA)) to help them grow, develop, and adapt to their changing environments [4,5]. Both ABA and methyl jasmonate (MeJA) can induce increases in cytosolic Ca^2+^ ([Ca^2+^]_cyt_) in plant guard cells and roots [6,7,8]. Using tobacco suspension cells transiently transformed with cytosolic- or nucleus-targeted apoaequorin, Walter et al. [9] found that both 12-oxophytodienoic acid, the precursor of JA, and JA itself could separately induce a transient increase in nuclear Ca^2+^ ([Ca^2+^]_nuc_) and [Ca^2+^]_cyt_ in a concentration-dependent manner. Krebs et al. [10] also demonstrated that external ATP could induce increases in [Ca^2+^]_nuc_ and [Ca^2+^]_cyt_ in *Arabidopsis thaliana* root cells and Nod factor-induced spikes in [Ca^2+^]_nuc_ and [Ca^2+^]_cyt_ in *Lotus japonicus* root hair cells. Additionally, osmotic and salt stresses can separately trigger mutually independent increases in [Ca^2+^]_nuc_ and [Ca^2+^]_cyt_ in *A. thaliana* roots; such increases are involved in specific gene expression, root growth, and lateral root development [11]. Therefore, deciphering the [Ca^2+^]_cyt_- and [Ca^2+^]_nuc_-regulated molecular networks and their crosstalk with hormone signalling pathways is essential for greater comprehension of Ca^2+^ signalling in plants.

Ca^2+^ signals are characterised as stimulus-specific signatures in terms of duration, amplitude, and frequency of [Ca^2+^] elevation [3]; they are sensed by intracellular Ca^2+^ sensor proteins composed of calmodulins (CaMs) and calmodulin-like proteins (CMLs) [12], Ca^2+^-dependent protein kinases (CPKs), CPK-related kinases (CRKs) [13], Ca^2+^- and Ca^2+^/CaM-dependent protein kinases (CCaMKs) [14,15], and calcineurin B-like proteins (CBLs), along with CBL-interacting protein kinases (CIPKs) [16]. Complex gene families encode these Ca^2+^ sensors; most possess classical EF-hand motifs, which allow for the binding of Ca^2+^ and triggering of Ca^2+^-dependent conformational changes within these proteins for signal transduction [17,18]. Throughout the *A. thaliana* genome, the *CaM/CML* family is represented by 6 *CaM* and 50 *CML* genes [19], the *CBL-CIPK* family has 10 *CBLs* and 25 *CIPKs* [20], and the *CPK/CRK* family is encoded by 34 *CPK* and 8 *CRK* genes [13]. In contrast to CaMs present in all eukaryotes, CMLs, CBLs-CIPKs, CCaMKs, and CPKs/CRKs—all of which form intricate signalling networks that enable phosphorylation events, changes in protein–protein interactions, and gene expression regulation—are mainly restricted to plants and some protists [21].

In plants, Ca^2+^ sensors can be classified into two major groups: sensor relays and sensor responders [2]. CaMs, CMLs, and CBLs do not have any known enzymatic functional domains; therefore, they are considered members of the sensor relay group. A notable exception is CaM7, which physically interacts with HY5 and directly binds the T/G- and E-box cis-acting elements in the *HY5* promoter to regulate its expression in *A. thaliana* [22,23]. In contrast, CPKs, CRKs, and CCaMKs are sensor responders; they contain one or more EF-hand motifs and a catalytic or functional domain, the activity of which is regulated by binding of Ca^2+^ to the EF-hand motifs. Elevated levels of [Ca^2+^]_nuc_ and [Ca^2+^]_cyt_ modulate gene expression through sensor relays or sensor responders via different mechanisms. CaMs and CMLs interact with DNA-binding proteins and regulate their activities, resulting in activation or repression of targeted gene expression. Furthermore, CPKs, CRKs, CCaMKs, and CBLs-CIPKs phosphorylate specific DNA-binding proteins to regulate gene expression [24]. In addition, a Ca^2+^-binding transcription factor, designated as AtNIG1 (*A. thaliana* NaCl-inducible gene 1), was initially identified as a basic helix–loop–helix-type transcription factor that contained an EF-hand motif and bound the E-box element (CANNTG) within the promoter regions of various salt stress-related genes [25]. CaM antagonists (e.g., N-(6-aminohexyl)-5-chloro-1-naphthelenesulphonamid-hydrochloride, trifluoperazine, fluphenazine-N-2-chloroethane dihydrochloride, and calmidazolium chloride) can induce increases of [Ca^2+^]_cyt_ in plant cells [26,27]. Therefore, using DNA microarrays to analyse early transcriptome changes, Kaplan and colleagues [27] revealed 230 [Ca^2+^]_cyt_-responsive genes. Furthermore, 269 [Ca^2+^]_cyt_-upregulated genes were identified in *A. thaliana* seedlings, using full genome microarray analysis [28]. However, [Ca^2+^]_nuc_-regulated genes have not been identified; the crosstalk between Ca^2+^ and hormone signalling pathways in response to diverse stresses and developmental cues remains elusive.

This study represents a comprehensive investigation of the *A. thaliana* transcriptome for [Ca^2+^]_nuc_- and [Ca^2+^]_cyt_-regulated genes in cytosolic-localised parvalbumin (*PV-NES*) and nucleosolic-localised parvalbumin (*PV-NLS*) transgenic *A. thaliana* seedlings in response to ABA and MeJA using full genome microarray analysis. This work revealed 244 [Ca^2+^]_cyt_- and [Ca^2+^]_nuc_-regulated ABA-responsive genes and 144 [Ca^2+^]_cyt_- and [Ca^2+^]_nuc_-regulated MeJA-responsive genes. Moreover, using systems biology approaches focusing on genes transcriptionally regulated by Ca^2+^ and hormones, we defined a highly reliable gene co-expression network among [Ca^2+^]_cyt_- and [Ca^2+^]_nuc_-regulated genes, Ca^2+^ signal-decoding proteins, and ABA/JA signalling pathway proteins to uncover dynamic subnetwork structures in response to hormone treatment in *A. thaliana*.

## 2. Materials and Methods

### 2.1. Plant Material and Growth Conditions

*A. thaliana* (Col-0 ecotype) plants were grown with a cycle of 16 h light (120 μmol m^−2^ s^−1^)/8 h dark at 22 °C and 60% relative humidity. T-DNA insertion mutants *cam1* (SALK_202076c), *gad1* (SALK_022227), and *cipk8* (SALK_139697c) were obtained from The *A. thaliana* Information Resource (https://www.arabidopsis.org/ (accessed on 6 September 2017)) (Figure S1A). After genotyping progeny from self-crossed plants by polymerase chain reaction (PCR) (Figure S1B) and assessing target gene transcription in seedlings by quantitative reverse transcription PCR (qRT-PCR) (Figure S1C), we obtained homozygous lines of *cam1*, *cipk8*, and *gad1*. The used primers are listed in Table S1.

*A. thaliana* seeds were surface-sterilised with 75% ethanol and plated on half-strength Murashige and Skoog (MS) salts, 2% sucrose, and 0.8% (*w/v*) agar at pH 5.8. After stratification at 4 °C in the dark for 3 days, the plates were transferred to a growth chamber under 16 h light (120 μmol m^−2^ s^−1^)/8 h dark at 22 °C for another 3 days. Seedlings were then transferred to half-strength MS plates containing 10 μM ABA or 50 μM MeJA, cultured for 5 additional days, and collected for RNA extraction. For the germination assay, seeds were plated on half-strength MS plates containing 0.5 μM ABA or 50 μM MeJA at 4 °C in the dark for 3 days. The plates were then transferred to a growth chamber for 3 days, and the seed germination rate was calculated.

### 2.2. Imaging of [Ca^2+^]_cyt_ and [Ca^2+^]_nuc_ in A. thaliana Roots

[Ca^2+^] measurements were performed in mature root sections of 1-week-old *A. thaliana* seedlings, as previously described [11]. Transgenic *A. thaliana* lines containing cytosolic-localised yellow Cameleon YC 3.6 (NES-YC3.6) or nuclear-localised yellow Cameleon YC 3.6 (NLS-YC3.6) were used to monitor the effects of ABA and MeJA on changes in [Ca^2+^]_cyt_ and [Ca^2+^]_nuc_ in root cells. After germination, *A. thaliana* seedlings were grown vertically on half-strength MS medium for 5–7 days; the roots were immobilised by overlaying 1% (*w/v*) low-melting-point agarose (AMRESCO, Dallas, TX, USA) in an Attofluor^®^ Cell Chamber (Invitrogen, Waltham, MA, USA). After a small tunnel in the agarose had been made to expose the root, 200 μL of bathing solution buffer (0.5× MS salt, 1% sucrose, 10 mM MES-KOH, pH 5.8) were applied to the chamber. ABA (10 µM) and MeJA (50 µM) in the same bathing solution were separately perfused as the stimulus into the chamber. The mean fluorescence resonance energy transfer values in response to different stimuli represented measurement of 20–30 root cells from at least nine independent seedlings, each of which included three to six root cells. Analyses of statistical significance were performed using the unpaired Student’s *t*-test with GraphPad Prism 5.0 (GraphPad Software, San Diego, CA, USA) and the results are presented as means ± standard deviations (SDs).

### 2.3. Stomatal Aperture Bioassay

Rosette leaves were detached from 3- to 4-week-old *A. thaliana* plants and floated in a solution containing 10 mM KCl, 2 mM CaCl_2_, and 10 mM MES-Tris at pH 6.15 for 2 h in a growth chamber under light (120 μmol m^−2^ s^−1^) at 22 °C. ABA (10 μM) and MeJA (50 μM) were separately added to the solutions for 2 h to assess stomatal closure, as previously described [29]. Stomatal apertures were imaged with a digital camera (DP72; Olympus Corp., Tokyo, Japan) attached to a fluorescence microscope (BX51; Olympus Corp.) and measured using DP2-BAW software (Olympus Corp.).

### 2.4. Total RNA Isolation and Analysis of Microarray Data

Two wild-type (WT) plants and three previously described independent *PV-NES* lines (1, 7, and 11) and *PV-NLS* transgenic lines (4, 5, and 8) [11] were used for microarray analysis. First, total RNA was extracted from seedlings that had been treated with 10 μM ABA or 50 μM MeJA for 5 days using the Eastep^®^ Super Total RNA Extraction Kit (Promega, Madison, WI, USA), in accordance with the manufacturer’s instructions. Approximately 2 μg of total RNA were reverse-transcribed into first-strand cDNA using the First-Strand cDNA Synthesis SuperMix (TransScript, Beijing, China). The cDNA was hybridised on an Affymetrix *A. thaliana* ATH1-121501 genome array using a GPL198 platform (CapitalBio Corporation, Beijing, China). All generated datasets are publicly available in the Gene Expression Omnibus database under the accession number GSE109611.

Files in .cel format were read into R and normalised using the RMA procedure. The cor function was used to calculate the Pearson correlation coefficient, represented by R. The R-value of duplicate samples ranged from 0.98 to 1, indicating high relevance and repeatability. Volcano plot analysis, with a fold-change >2.0 and a false discovery rate-corrected *p*-value < 0.05, according to Student’s *t*-test, was performed to identify differentially expressed genes; such genes were visualised using Venn diagrams and heatmaps.

### 2.5. Construction of the A. thaliana Gene Co-Expression Network

Seven open or published *A. thaliana* chip datasets regarding ABA and JA hormone processing were collected from the Affymetrix GPL198 platform and downloaded from the Gene Expression Omnibus database on the National Center for Biotechnology Information website: GSE12715, GSE45662 [30], GSE84446, GSE39384 [31], GSE7432 [32], GSE5620 [33], and GSE109611. In accordance with a previously published method [34], R software was used to pre-process the data of each chip, mainly in terms of removing disproportionately low mean expression genes or genes with poor correlations. Finally, the dataset contained 13,083 genes in 165 samples. Then, the weighted gene co-expression network analysis algorithm in R software was used to construct the *A. thaliana* co-expression network. This algorithm can generate functional modules in various situations [35,36]. To construct the co-expression network, the weight value power selected in this study was 12; the corresponding Pearson correlation coefficient was approximately 0.9. Cytoscape_V3.2.1 was used to display the co-expression network results.

### 2.6. Firefly Luciferase Complementation Imaging (LCI) Assay

The LCI assay was conducted as previously described [37]. Coding sequences of glutamate decarboxylase 1 (GAD1), calmodulin 1 (CAM1), and calcineurin B-like protein-interacting protein kinase 8 (CIPK8) were inserted into the pCAMBIA-nLUC or pCAMBIA-cLUC vector. The Ca^2+^-regulated hormone response genes were cloned into the pCAMBIA-cLUC vector. The constructed plasmids and conjugative P19 plasmid were separately transformed into Agrobacterium GV3101 strains. A single colony was inoculated in the corresponding resistant YEB liquid medium and cultured overnight at 28 °C and 200 rpm. After centrifugation for 2 min at 12,000 rpm, the medium was discarded. The pellet was washed five times with tobacco transformation buffer (10 mM MES, 10 mM MgCl_2_, pH 5.6), resuspended with tobacco transformation buffer containing 0.1 mM acetosyringone, and infiltrated into the leaves of 3-week-old *Nicotiana benthamiana* (16 h day/8 h night, 25 °C) with pairs of nLUC and cLUC vectors. After 2–3 days, the luciferase assay substrate (Promega) was infiltrated into the leaf and reaction imaging was immediately captured by a low-light cooled charge-coupled device imaging system (Bio-Rad Laboratories, Inc., Hercules, CA, USA). The experiments were repeated at least three times.

### 2.7. qRT-PCR

Four-day-old seedlings were transferred to half-strength MS medium containing 10 μM ABA or 50 μM MeJA for 6 h, followed by RNA extraction and first-strand cDNA as described in Section 2.4. qRT-PCR was performed with Power SYBR^®^ Green PCR Master Mix (TransStart, Beijing, China) on a 7500 Fast Real-Time PCR System (Applied Biosystems, Foster City, CA, USA). Value changes of more than two-fold (>2 or <0.5) were considered to indicate the induction or repression of gene expression. All primers used in this study are listed in Table S1. The *A*. *thaliana actin2* gene served as an internal control. At least three independent biological replicates were performed.

### 2.8. Statistical Analysis

Data analyses were carried out using the Data Processing System [38]. Data are shown as means ± SDs of at least three independent experiments. Statistical comparisons were performed using one-way or two-way analysis of variance with Student’s *t*-test to identify significant differences among group means; *p* < 0.05 was considered to indicate statistical significance.

## 3. Results

### 3.1. Both ABA and MeJA Triggered [Ca^2+^]_cyt_ and [Ca^2+^]_nuc_ Increases in A. thaliana Roots

**Figure 1:**
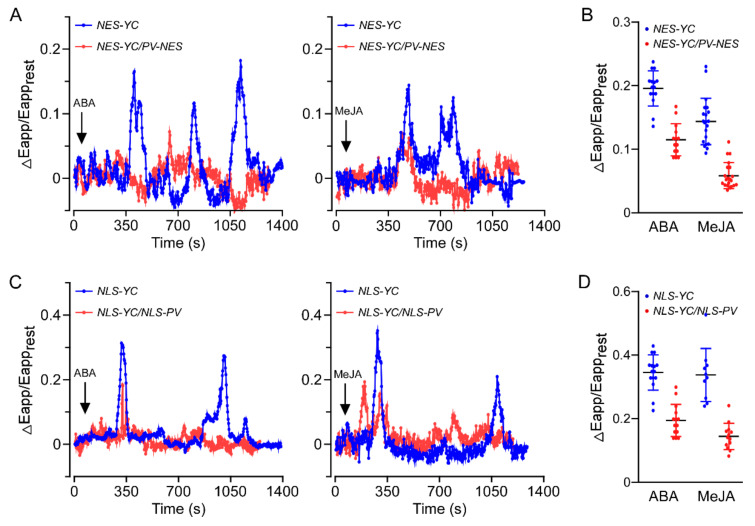
Impaired [Ca^2+^]_cyt_ and [Ca^2+^]_nuc_ increases in responses to ABA and MeJA treatment in root cells of *PV-NES* and *PV-*NLS transgenic *A. thaliana* lines, respectively. (**A**,**B**) Changes in [Ca^2+^]_cyt_ in the roots of 6-day-old WT and PV-NES plants triggered by ABA (50 μM) or MeJA (1 mM). The dynamics of [Ca^2+^]_cyt_ are shown as changes in the apparent fluorescence resonance energy transfer efficiency of NES-YC3.6. Typical traces (**A**) and [Ca^2+^]_cyt_ peak value comparisons (**B**) are shown (*n* = 20 cells from 8 different seedlings). (**C**,**D**) Changes in [Ca^2+^]_nuc_ in the roots of 6-day-old WT and PV-NLS plants triggered by ABA (50 μM) or MeJA (1 mM). The dynamics of [Ca^2+^]_cyt_ are shown as changes in the apparent fluorescence resonance energy transfer efficiency of NES-YC3.6. Typical traces (**C**) and [Ca^2+^]_cyt_ peak value comparisons (**D**) are shown (*n* = 20 cells from 8 different seedlings). In (**B**,**D**), black lines represent the mean value; blue and red lines represent the standard error (*p* < 0.001).

Transgenic *A. thaliana* lines containing cytosolic-localised yellow Cameleon YC 3.6 (NES-YC3.6) or nuclear-localised yellow Cameleon YC 3.6 (NLS-YC3.6) were used to monitor the effects of ABA and MeJA on changes in [Ca^2+^]_cyt_ and [Ca^2+^]_nuc_ in root cells. [Ca^2+^] measurements were performed in mature root sections of 1-week-old *A. thaliana* seedlings, as previously described [11]. Consistent with previous results from plant stomata [6,39], we demonstrated that both ABA and MeJA triggered [Ca^2+^]_cyt_ elevations in *A. thaliana* roots (Figure 1A,B). Moreover, ABA and MeJA induced [Ca^2+^]_nuc_ increases in *A. thaliana* root cells (Figure 1C,D), indicating that [Ca^2+^]_nuc_, similar to [Ca^2+^]_cyt_, participates in ABA and JA signalling within plants. In addition, the elevations of [Ca^2+^]_cyt_ and [Ca^2+^]_nuc_ elicited by ABA or MeJA were impaired in root cells of *PV-NES/NES-YC3.6* and *PV-NLS/NLS-YC3.6* plants, respectively, compared with the WT (Figure 1).

### 3.2. Elevated [Ca^2+^]_cyt_ and [Ca^2+^]_nuc_ Contributed to Significant Changes in ABA- or MeJA-Responsive Gene Expression Profiles in A. thaliana

**Figure 2:**
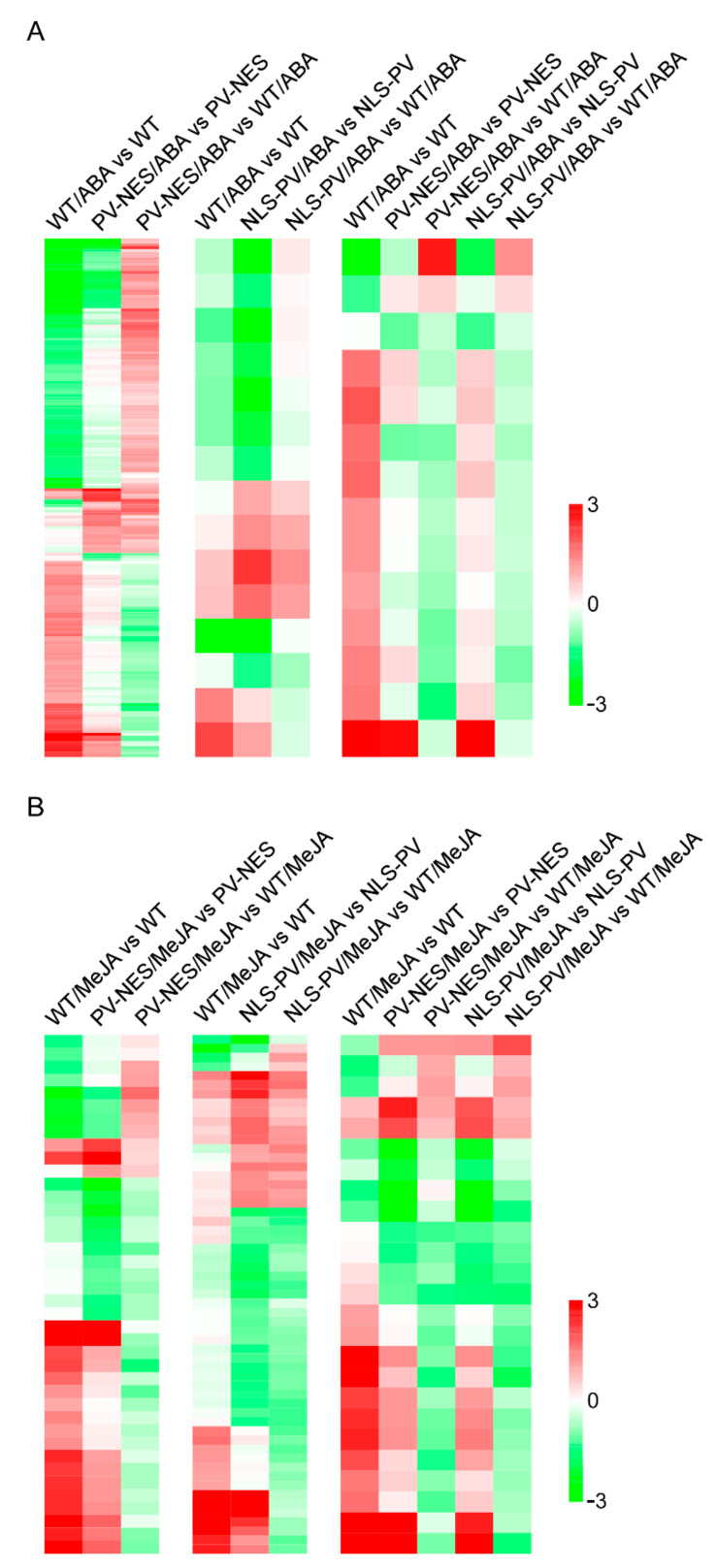
[Ca^2+^]_cyt_- and [Ca^2+^]_nuc_-regulated phytohormone-responsive genes. Significantly differentially expressed gene clusters in WT, *PV-NES,* and *PV-NLS* transgenic lines in response to ABA (**A**) and MeJA (**B**) treatment. The left column indicates [Ca^2+^]_cyt_-regulated ABA- or MeJA-responsive genes. The middle column indicates [Ca^2+^]_nuc_-regulated ABA- or MeJA-responsive genes. The right column indicates [Ca^2+^]_cyt_- and [Ca^2+^]_nuc_-regulated ABA- or MeJA-responsive genes. Microarray comparison results represented by each column are indicated at the top of the figure. Each row represents one gene; colours represent changes in gene expression levels as indicated. Quantitative data for each gene are shown in Tables S2 and S3.

To further elucidate the roles of [Ca^2+^]_cyt_ and [Ca^2+^]_nuc_ in gene expression in *A. thaliana*, genome microarray analyses were performed in *PV-NES* and *PV-NLS* seedlings in response to long-term treatment with ABA or MeJA. Three lines of each transgenic plant and two WT plants were used in the microarray analysis experiments; the datasets were deposited into the Gene Expression Omnibus database with the accession number GSE109611. Using the parameters of a 2.0-fold change and a *p*-value < 0.05, we identified 1001, 667, and 889 ABA-responsive genes in WT, *PV-NES*, and *PV-NLS* lines, respectively (Figure S2A–C); 1145, 1095, and 1262 MeJA-responsive genes were identified in WT, *PV-NES*, and *PV-NLS* lines, respectively (Figure S2D–F). Then, we identified 193 [Ca^2+^]_cyt_-regulated ABA-responsive genes, 15 [Ca^2+^]_nuc_-regulated ABA-responsive genes, and 14 [Ca^2+^]_cyt_- and [Ca^2+^]_nuc_-co-regulated ABA-responsive genes between WT and *PV-NES* or *PV-NLS* plants (Figure 2A and Figure S3 and Table S2); we also identified 40 [Ca^2+^]_cyt_-regulated MeJA-responsive genes, 57 [Ca^2+^]_nuc_-regulated MeJA-responsive genes, and 25 [Ca^2+^]_cyt_- and [Ca^2+^]_nuc_-co-regulated MeJA-responsive genes between WT and *PV-NES* or *PV-NLS* plants (Figure 2B and Figure S3 and Table S3). In addition, among these differentially expressed [Ca^2+^]_cyt_- or [Ca^2+^]_nuc_-regulated hormone-responsive genes, we identified eight [Ca^2+^]_cyt_-regulated ABA- and MeJA-responsive genes; two [Ca^2+^]_nuc_-regulated ABA- and MeJA-responsive genes; seven [Ca^2+^]_cyt_-regulated ABA-responsive and [Ca^2+^]_cyt_- and [Ca^2+^]_nuc_-co-regulated MeJA-responsive genes; two [Ca^2+^]_cyt_-regulated ABA-responsive and [Ca^2+^]_nuc_-regulated MeJA-responsive genes; one [Ca^2+^]_cyt_- and [Ca^2+^]_nuc_-co-regulated ABA-responsive and [Ca^2+^]_nuc_-regulated MeJA-responsive gene; one [Ca^2+^]_cyt_-regulated MeJA-responsive and [Ca^2+^]_nuc_-regulated ABA and MeJA-responsive gene; and one [Ca^2+^]_cyt_- and [Ca^2+^]_nuc_-co-regulated ABA and MeJA-responsive gene (Figure S3). These results suggest that both cytosolic and nucleosolic Ca^2+^ signals are involved in hormone-regulated gene expression and network crosstalk in *A. thaliana*.

### 3.3. Integrative Co-Expression Analysis to Identify Hub Genes within Calcium-Regulated Transcriptional Modules

To further characterise the roles of calcium in phytohormone-regulated gene expression, we generated integrative co-expression networks of [Ca^2+^]-regulated hormone-responsive genes with Ca^2+^ signal-decoding genes and ABA or JA pathway proteins. First, we collected *A. thaliana* microarray datasets that were used in the Affymetrix platform GPL198 analysis, filtered outliers from the data using a published method [34], and compiled microarray datasets containing 165 samples and 13,083 genes (Table S4); these covered approximately half of the coding genes in the *A. thaliana* genome. Subsequently, we constructed a co-expression network map by calculating the Pearson correlation coefficient among the expression values of different genes in accordance with the standard WGCNA procedure; we identified modules with biologically correlated genes. The power value was set to 12 and the Pearson correlation coefficient was set to >0.9, indicating a high correlation; the most highly connected genes within each module, designated as ‘hubs’, could be key regulators in response to ABA and/or MeJA in *A. thaliana*.

**Figure 3:**
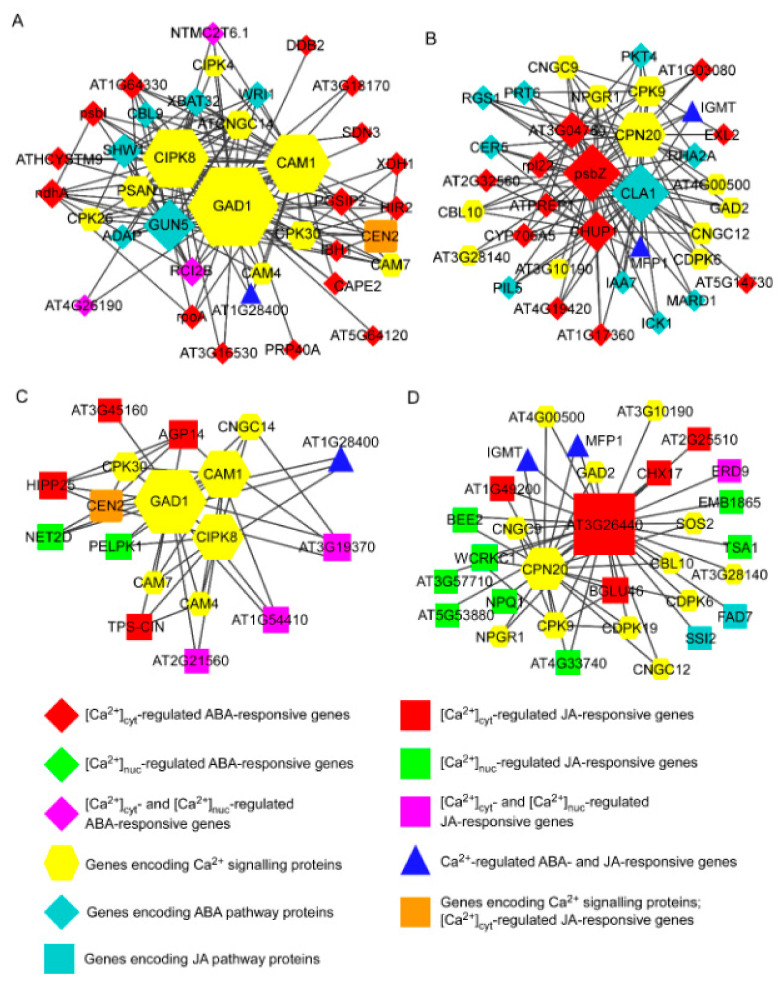
Calcium-mediated phytohormone-associated gene co-expression networks. (**A**,**B**) Sub-models with distinct central genes and their co-expression partners among [Ca^2+^]_cyt_- and [Ca^2+^]_nuc_-regulated ABA-responsive genes, genes encoding calcium signalling proteins, and genes encoding ABA pathway proteins. (**C**,**D**) Sub-models with distinct central genes and their co-expression partners among [Ca^2+^]_cyt_- and [Ca^2+^]_nuc_-regulated MeJA-responsive genes, genes encoding calcium signalling proteins, and genes encoding JA pathway proteins. Different colours and shapes indicate distinct types of proteins. Different genes in the models are listed in Table S8.

Next, we placed Ca^2+^-regulated ABA-responsive genes, calcium signal-decoding genes (Table S5), and ABA pathway member genes (Table S6) into the constructed *A. thaliana* gene co-expression database to obtain a Ca^2+^-regulated ABA signalling co-expression network (Figure 3A,B, Table S7). Similarly, we placed Ca^2+^-regulated MeJA-responsive genes, calcium decoding protein genes, and JA pathway member genes into the constructed gene co-expression database to obtain a Ca^2+^-regulated JA signalling co-expression network (Figure 3C,D, Table S7). We found that three calcium signal-decoding genes—*calmodulin 1* (*CAM1*), *calcineurin B-like protein-interacting protein kinase 8* (*CIPK8*), and *glutamate decarboxylase 1* (*GAD1*)—were hub genes in the main submodules of Ca^2+^-regulated ABA and JA signalling co-expression networks (Figure 3A,C). In these two modules, *CAM1, CIPK8*, and *GAD1* were co-expressed with [Ca^2+^]_cyt_-regulated ABA-responsive genes such as *AT1G64330*, *NAD(P)H-quinone oxidoreductase subunit 1* (*NDHA*), *HYPERSENSITIVE INDUCED REACTION 2* (*HIR2*), *CAPE2*, *PGSIP2*, and *PSBI*; [Ca^2+^]_cyt_- and [Ca^2+^]_nuc_-co-regulated ABA-responsive genes such as *NTMC2T6.1*, *RCI2B*, and *AT4G26190*; [Ca^2+^]_cyt_-regulated JA-responsive genes such as *AT3G45160*, *AGP14*, and *HIPP25*; [Ca^2+^]_nuc_-regulated JA-responsive genes such as *PELPK1* and *NET2D*; [Ca^2+^]_cyt_- and [Ca^2+^]_nuc_-co-regulated JA-responsive genes such as *AT3G19370*, *AT1G54410*, and *AT2G21560*; and the [Ca^2+^]_cyt_-regulated ABA-responsive and [Ca^2+^]_cyt_- and [Ca^2+^]_nuc_-co-regulated JA-responsive gene AT1G28400 (Figure 3A,C). In two other submodules, the calcium decoding protein gene *CPN20* co-expressed with *PSBZ*, a [Ca^2+^]_cyt_-regulated ABA-responsive gene, and *CLA1*, an ABA pathway member, constituted the hub genes of a Ca^2+^-regulated ABA signalling co-expression network (Figure 3B). Similarly, *CPN20* co-expressed with *AT3G26440*, a [Ca^2+^]_cyt_-regulated JA-responsive gene, was identified as a hub component in a Ca^2+^-regulated JA signalling co-expression network (Figure 3D). These results indicated that these hub genes, *CAM1*, *CIPK8*, *GAD1*, *and CPN20*, have important regulatory roles in cytosolic and nucleosolic calcium-mediated hormone-responsive gene expression.

### 3.4. Interactions among the Hub Proteins CAM1, CIPK8, and GAD1 and with Proteins Encoded by Ca^2+^-Regulated Hormone-Responsive Genes

**Figure 4:**
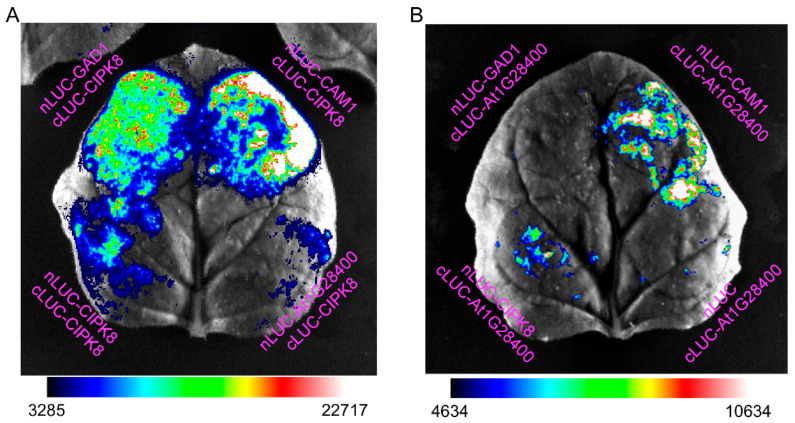
Interactions among four hub genes in tobacco leaves, as determined by LCI assays. (**A**) The interaction of CIPK8 with CAM1, GAD1, CIPK8 or At1G28400. (**B**) The interaction of At1G28400 with CAM1, GAD1, or CIPK8. Tobacco leaves were transformed with constructs encoding full-length *GAD1*, *CAM1*, *CIPK8*, and *At1G28400* fused with the C- or N-terminus of luciferase (c- or n-LUC). Empty vectors were used as negative controls. Luminance intensity indicates an interaction between any two proteins. The experiments were repeated three times and a representative read-out is shown.

We next performed the LCI assay in the leaves of 3-week-old *N*. *benthamiana* to detect interactions among the three hub proteins, as well as interactions with proteins encoded by Ca^2+^-regulated hormone-responsive genes. First, we showed an interaction of CIPK8 with CAM1, GAD1, and itself; we also showed an interaction of the protein encoded by AT1G28400, a [Ca^2+^]_cyt_-regulated ABA-responsive and [Ca^2+^]_cyt_- and [Ca^2+^]_nuc_-co-regulated JA-responsive gene, with CAM1, but not with GAD1 and CIPK8 (Figure 4). Interactions between the encoding proteins of several Ca^2+^-regulated hormone-responsive genes and each of the three hub genes are summarised in Table 1; the interaction between CAM1 and GAD1 was used as a positive control. In the [Ca^2+^]_cyt_-regulated ABA-responsive genes *AT1G64330*, *NDHA*, *HIR2*, *CAPE2*, *PGSIP2*, and *PSBI*, we observed interactions of AT1G64330 with GAD1 or CAM1, PSBI with CAM1 or CIPK8, and PGSIP2 with CAM1 (Figures S4 and S5). The encoded protein of *AT3G45160*, a [Ca^2+^]_cyt_-regulated JA-responsive gene, interacted with GAD1 and CAM1, but not CIPK8 (Figure S5). In the [Ca^2+^]_cyt_- and [Ca^2+^]_nuc_-co-regulated ABA-responsive genes *NTMC2T6.1 and RCI2B*, we observed interactions of RCI2B with GAD1 or CAM1, and NTMC2T6.1 with CAM1 (Figure S6). In the [Ca^2+^]_cyt_- and [Ca^2+^]_nuc_-co-regulated JA-responsive genes *AT3G19370* and *AT1G54410*, we observed interactions of AT3G19370 with GAD1 or CAM1; there were no interactions between AT1G54410 and any of the three hub proteins (Figure S7). These results provide clues for dissecting the mechanism underlying hub protein-mediated regulation of the expression of ABA and JA responsive genes.

### 3.5. CAM1 and CIPK8 Are Required for MeJA-Induced Stomatal Closure and Are Associated with ABA-Inhibited Seed Germination

**Figure 5:**
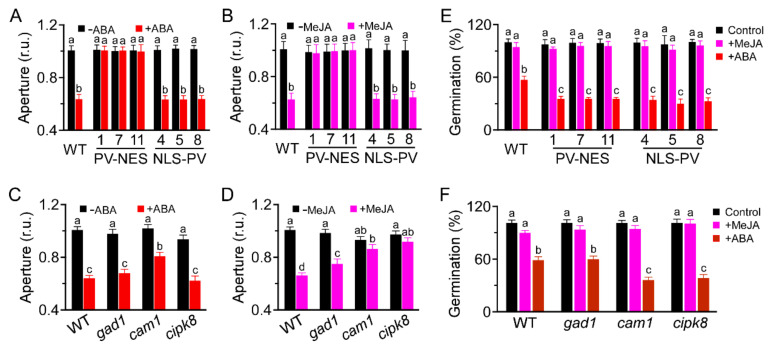
Impacts of [Ca^2+^]_cyt_ and [Ca^2+^]_nuc_ on ABA- or MeJA-induced stomatal closure and seed germination. (**A**–**D**) Stomatal apertures (W/L, width/length) of rosette leaves from 3-week-old WT, *PV-NES*, and *PV-NLS* plants, and *gad1*, *cam1*, and *cipk8* plants, were measured after treatment with 10 μM ABA (**A**,**C**) or 50 μM MeJA (**B**,**D**). r.u. indicates relative units. Data from six independent experiments and a total of 120 stomata are shown; data are shown as means ± SDs. Different letters indicate statistical significance at *p* < 0.05 (Student’s *t*-test). (**E**,**F**) Seed germination of WT, *PV-NES*, and *PV-NLS* transgenic lines (**E**) and of WT, *gad1*, *cam1*, and *cipk8* plants (**F**) in response to ABA or JA treatment. Seeds were plated on half-strength MS plates containing ABA (10 μM) or MeJA (50 μM) for 3 days at 4 °C in the dark, then transferred to a growth chamber under 16 h light (120 μmol m^−2^ s^−1^)/8 h dark at 22 °C for another 3 days. Error bars represent SDs; n = 600 seeds. Different letters indicate significant differences at *p* < 0.05 (Student’s *t*-test).

We monitored the roles of [Ca^2+^]_cyt_ and [Ca^2+^]_nuc_ in hormone-induced stomatal closure in *A. thaliana*. Both ABA- and MeJA-induced stomatal closure were severely impaired in *PV-NES* plants, but not in *PV-NLS* plants (Figure 5A,B), indicating that ABA- and MeJA-induced stomatal closure requires [Ca^2+^]_cyt_, but not [Ca^2+^]_nuc_. Furthermore, ABA-induced stomatal closure was impaired in *cam1*, but not *gad1* and *cipk8* (Figure 5C); MeJA-induced stomatal closure was impaired in *cam1* and *cipk8*, but not *gad1* (Figure 5D). These results suggest that CAM1 is required for ABA- and MeJA-induced stomatal closure, while CIPK8 is essential for MeJA-induced stomatal closure. In addition, ABA-inhibited seed germination was more severe in both *PV-NES* and *PV-NLS* plants than in WT plants (Figure 5E). Similarly, ABA-inhibited seed germination was more severe in *cam1* and *cipk8* than in *gad1* (Figure 5F). However, MeJA had no effect on seed germination in any of the tested plants (Figure 5E,F). These results indicate that CAM1 and CIPK8 regulate ABA-inhibited seed germination.

### 3.6. CAM1, CIPK8, and GAD1 Regulate the Transcription of [Ca^2+^]-Regulated Genes in Response to ABA or MeJA Treatment

**Figure 6:**
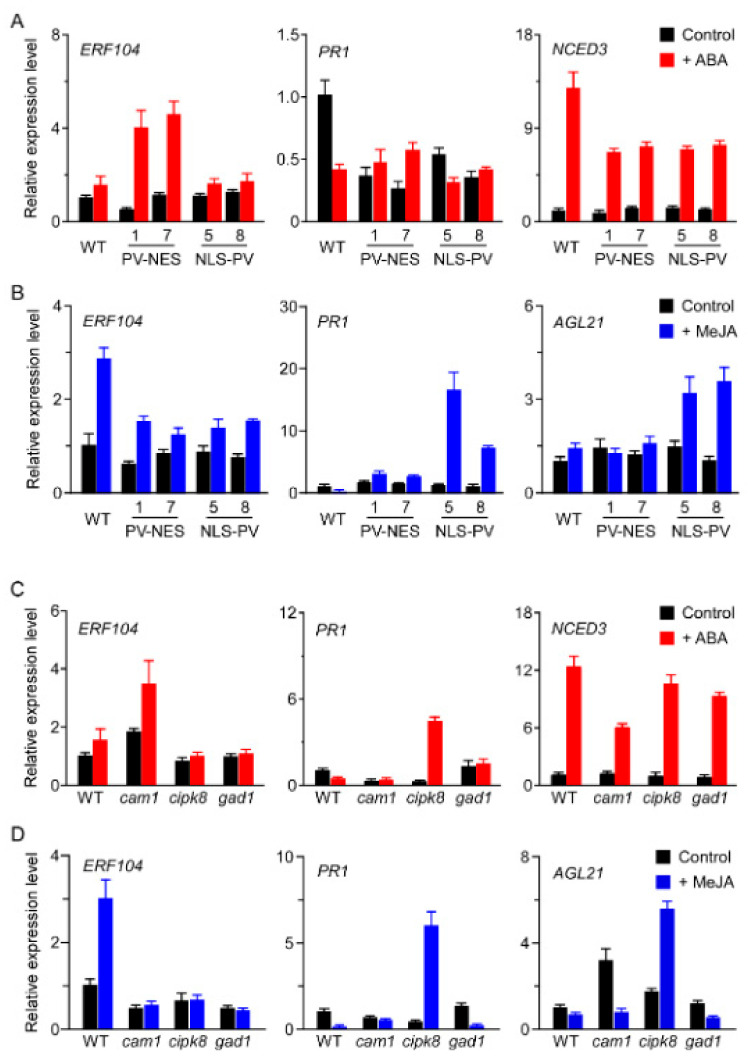
Expression patterns of several [Ca^2+^]-regulated genes were detected by qRT-PCR in *A. thaliana* seedlings. (**A**,**B**) Transcript abundances of *ERF104*, *PR1*, and *NCED3* in response to ABA (10 μM) or MeJA (50 μM) in WT, *PV-NES*, and *PV-NLS* seedlings. (**C**,**D**) Transcript abundances of *ERF104*, *PR1*, and *NCED3* in response to ABA (10 μM) and abundances of *ERF104*, *PR1*, and *AGL21* in response to MeJA (50 μM) in WT, *cam1*, *cipk8*, and *gad1* seedlings. Expression levels of target genes were normalised to the expression level of *actin2*, and the relative value of the WT was set to 1.0. Data are shown as means ± SDs of three biological replicates.

Here, we found that *NCED3* was a [Ca^2+^]_cyt_-regulated ABA-responsive gene, *ERF104* was a [Ca^2+^]_cyt_-regulated ABA- and MeJA-responsive gene, *PR1* was a [Ca^2+^]_nuc_-regulated ABA- and MeJA-responsive gene, and *AGL21* was a [Ca^2+^]_cyt_- and [Ca^2+^]_nuc_-regulated MeJA-responsive gene (Tables S2 and S3). Therefore, we monitored the expression patterns of these [Ca^2+^]-regulated genes in different *A. thaliana* plants treated with ABA or MeJA for 6 h by qRT-PCR. First, we found that *ERF104* was induced by ABA only in *PV-NES* plants and by MeJA only in WT plants (Figure 6A,B), consistent with our microarray data. In addition, *ERF104* was induced by ABA in *cam1*, but not in *cipk8* and *gad1*; it was not induced by MeJA in these three mutant lines (Figure 6C,D). Thus, CAM1 is required for ABA-induced expression of *ERF104*; CAM1, CIPK8, and GAD1 positively regulate MeJA-induced expression of *ERF104*. Second, we found that *PR1* was downregulated by ABA and MeJA in WT plants, but strongly upregulated by MeJA in *PV-NLS* plants (Figure 6A,B). Furthermore, *PR1* expression was activated by ABA and MeJA in *cipk8*, but the effects were less robust in *cam1* and *gad1* (Figure 6C,D). Third, *NCED3* was activated by ABA in WT plants; however, this type of activation was decreased in *PV-NES, PV-NLS*, and *cam1* lines (Figure 6A,C). Finally, *AGL21* was induced by MeJA in *PV-NLS* and *cipk8* plants, but not in WT, *PV-NES*, or *gad1* plants (Figure 6B,D). These results suggested that CAM1, CIPK8, and GAD1 act as key regulators that modify the expression patterns of calcium-regulated genes in response to ABA and MeJA in *A. thaliana* seedlings.

## 4. Discussion

Ca^2+^ serves as a vital secondary messenger in the mediation and integration of multiple hormone signalling pathways that specify cell-signalling information during plant growth and development. Therefore, it is challenging to elucidate the complexity of hormone and Ca^2+^ crosstalk; such analysis requires a combined approach that involves both experimental measurements and systems-level modelling. A prior study revealed 269 [Ca^2+^]_cyt_-upregulated genes in *A. thaliana* seedlings that responded to three specific types of [Ca^2+^]_cyt_ elevations elicited by artificial electrical stimulation [28]. However, the identification of [Ca^2+^]-responsive genes under physiological conditions in plants remains challenging. Our recent study demonstrated that both osmotic- and salt-stress-induced [Ca^2+^]_cyt_ and [Ca^2+^]_nuc_ increases were impaired in the roots of transgenic *A. thaliana* lines containing *PV-NES* or *PV-NLS*, respectively [11], thus providing a powerful tool for the establishment of an interaction landscape of hormone and Ca^2+^ signalling in *A. thaliana*. Here, we identified 244 [Ca^2+^]_cyt_- and [Ca^2+^]_nuc_-regulated ABA-responsive and 144 [Ca^2+^]_cyt_- and [Ca^2+^]_nuc_-regulated MeJA-responsive genes in *A. thaliana* seedlings using full genome microarray analysis; we found that 22 genes overlapped. Unlike other Ca^2+^-based transcriptome studies, we confined our conditions to a long duration of exposure to ABA or MeJA; this facilitated enrichment of late transcriptional events. Through co-expression network analysis, we unveiled two common modules among [Ca^2+^]_cyt_- and [Ca^2+^]_nuc_-regulated hormone-responsive genes, Ca^2+^ signal-decoding genes, and ABA/JA signalling pathway genes; our results provide valuable clues for use in exploring novel functions of known genes or potential functions of unknown genes, while enabling the dissection of crosslinks among Ca^2+^ signalling and hormonal pathways via co-expression networks.

Previous studies showed that cytosolic and nucleosolic calcium increases in response to various stimuli are mutually independent in animal [40] and plant cells [11]. Moreover, gene expression patterns regulated by nuclear calcium are also independent of cytosolic calcium in animal cells. For example, signalling pathways activated by [Ca^2+^]_cyt_ target the serum-response element, whereas [Ca^2+^]_nuc_ increases are critical for cyclic AMP response element-dependent calcium-activated transcription in hippocampal neurons [41]. However, Thompson et al. [42] found that [Ca^2+^]_nuc_, but not [Ca^2+^]_cyt_, negatively regulates the activity of transcription enhancer factor in Chinese hamster ovary (CHO) cells. In plants, [Ca^2+^]_nuc_ oscillations in response to Nod factor treatment are mediated by three nuclear-localised cyclic nucleotide-gated channels (CNGC15a/b/c); such oscillations are required for the establishment of symbiosis by nitrogen-fixing rhizobial bacteria in *Medicago truncatula* [43]. Here, we characterised [Ca^2+^]_cyt_-activated/repressed, [Ca^2+^]_nuc_-activated/repressed, and [Ca^2+^]_cyt_- and [Ca^2+^]_nuc_-co-regulated genes in response to ABA and MeJA treatment in *A. thaliana* seedlings. Furthermore, some genes were shared among the [Ca^2+^]-regulated ABA-responsive and [Ca^2+^]-regulated MeJA-responsive genes, indicating that both cytosolic and nucleosolic calcium are involved in the transcriptional regulation triggered by ABA or MeJA treatment in *A. thaliana*. Notably, we found that four calcium signal-decoding genes, *CAM1*, *GAD1*, *CIPK8*, and *CPN20*, were hub genes in two modules of Ca^2+^-regulated ABA and JA signalling co-expression networks. Additionally, we found the direct interactions among hub genes and Ca^2+^-regulated hormone-responsive genes with different combinations in LCI assays. These results provide important clues concerning hub genes *CAM1*, *GAD1*, *CIPK8*, and *CPN20* that mediate crosstalk between Ca signalling and hormone pathways in *A. thaliana*, suggesting the implications for other plants or crops under biotic and abiotic stress [44,45].

An intriguing finding was that the chloroplast genome encoded several [Ca^2+^]_cyt_-activated ABA-responsive genes, including *PSBI*, *YCF9*, *PSBJ*, *RPOA*, *RPL14*, *RPS3*, *RPL22*, *RPS19*, *YCF1.1*, *PSAC*, *ndhE*, and *ndhA*, as well as one [Ca^2+^]_cyt_- and [Ca^2+^]_nuc_-activated ABA-responsive gene, *PSBT*, and one [Ca^2+^]_nuc_-repressed MeJA-responsive gene, *PSBD*. A previous study showed that the chloroplast can function as a sensor for environmental stimuli, such as drought stress, and initiate signals that regulate nuclear gene expression [46]. Plastid-derived signals that target the regulation of nuclear gene expression are considered retrograde signals [47]; nucleus to plastid signalling is considered an anterograde pathway [48]. Wang et al. [49] showed that many genes related to drought stress responses, ABA metabolism, chloroplast biogenesis, and chlorophyll degradation are strongly expressed at early time points, followed by gradual decreases in induction or even suppression at later time points, during long-term ABA treatment in *A. thaliana*. Here, we found that *PSB1* and *ndhA* were co-expressed with hub genes *CAM1*, *CIPK8*, and *GAD1,* while *RPOA* was co-expressed with *CAM1* and *GAD1* in the Ca^2+^-regulated ABA signalling co-expression network. In addition, LCI assays showed that PSB1 interacts directly with CAM1 and CIPK8. These results indicated that the ABA-induced increase in [Ca^2+^]_cyt_, but not in [Ca^2+^]_nuc_, has a vital role in combining and coordinating the expression patterns of nuclear and plastid genes for bi-directional communication between chloroplasts and the nucleus [50].

Crosstalk between JA and ABA signalling through interactions of ABA receptor PYRABACTIN RESISTANCE1-Like proteins (PYLs) and JASMONATE ZIM DOMAIN (JAZ) proteins, transcription inhibitors of JA signalling, is known to coordinate the balance between plant growth and defence resistance [51]. Here, we revealed a new crosstalk mechanism between JA and ABA signalling via [Ca^2+^]_cyt_ and [Ca^2+^]_nuc_. We first showed that both ABA and MeJA induce [Ca^2+^]_cyt_ and [Ca^2+^]_nuc_ increases in *A. thaliana* roots; ABA- and MeJA-induced stomatal closure is impaired in *PV-NES*, but not *PV-NLS*, plants. In addition, ABA- and MeJA-induced stomatal closure is impaired in *cam1* plants. These results indicated that CAM1 perceives ABA- and MeJA-induced increases in [Ca^2+^]_cyt_ to regulate stomatal closure in *A. thaliana*. Hossain et al. [52] showed that MeJA-induced, but not ABA-induced, [Ca^2+^]_cyt_ elevation and stomatal closure are disrupted in the ABA-deficient mutant *aba2-2* or by treatment with the ABA synthetic inhibitor fluridon in WT plants, consistent with our results in *cipk8* plants; thus, CIPK8 presumably regulates ABA biosynthesis in *A. thaliana*. Furthermore, we found that ABA-inhibited seed germination is more sensitive in *PV-NES*, *PV-NLS*, *cam1*, and *cipk8* plants than in WT plants. These results indicated that both *CAM1* and *CIPK8* participate in cross-talk during ABA- and JA-regulated biological processes in *A. thaliana*. Using LCI assays, we showed that CIPK8 interacts with CAM1; many Ca^2+^-regulated ABA- and JA-responsive genes, such as ERF104, PR1, and AGL21, exhibit various expression patterns in *cam1* and *cipk8* plants. These results suggested that the CAM1–CIPK8 complex could be a connection between the ABA and JA signalling pathways. Further genetic and molecular studies are needed to explore this potential connection.

## 5. Conclusions

Here, we found that *PV-NES* and *PV-NLS* separately impair ABA- and MeJA-induced increases in [Ca^2+^]_cyt_ or [Ca^2+^]_nuc_. We also identified 244 [Ca^2+^]_cyt_- and [Ca^2+^]_nuc_-regulated ABA-responsive and 144 [Ca^2+^]_cyt_- and [Ca^2+^]_nuc_-regulated MeJA-responsive genes using an *A. thaliana* genome oligo array. Moreover, gene co-expression network analysis identified four Ca^2+^ signal-decoding genes, *CAM1*, *CIPK8*, *GAD1*, *and CPN20*, as hub genes in two [Ca^2+^]-regulated ABA/JA-responsive gene co-expression modules. Finally, LCI assays, phenotypic observations, and gene expression analyses showed that the CAM1–CIPK8 complex is a key regulator of stomatal movement, seed germination, and expression of [Ca^2+^]-regulated hormone-responsive genes in response to hormonal signals in *A. thaliana*.

## Figures and Tables

**Table 1:** Protein interactions among three hub genes and several [Ca^2+^]-regulated hormone-responsive genes detected by LCI assay (Figures S4–S7).

		nLUC-GAD1	nLUC-CAM1	nLUC-CIPK8
[Ca^2+^]_cyt_-regulated ABA-responsive genes	cLUC-NDHA	−	−	−
	cLUC-PSBI	−	+	+
	cLUC-PGSIP2	−	+	−
	cLUC-CAPE2	−	−	−
	cLUC-AT1G64330	+	+	−
	cLUC-HIR2	−	−	−
[Ca^2+^]_cyt_-regulated JA-responsive genes	cLUC-AT3G45160	+	+	−
[Ca^2+^]_cyt_- and [Ca^2+^]_nuc_-regulated ABA-responsive genes	cLUC-RCI2B	+	+	−
	cLUC-NTMC2T6.1	−	+	−
[Ca^2+^]_cyt_- and [Ca^2+^]_nuc_-regulated JA-responsive genes	cLUC-AT1G54410	−	−	−
	cLUC-AT3G19370	+	+	−

+ indicates interaction; − indicates no interaction.

## Data Availability

Not applicable.

## Supplementary Materials

The following are available online at https://www.mdpi.com/article/10.3390/genes13030524/s1, Figure S1: Characterisation of *A. thaliana* T-DNA insertion mutants *cam1*, *cipk8*, and *gad1*, Figure S2: Volcano plots of the total gene expression profiles of WT, *PV-NES*, and *PV-NLS* transgenic lines after treatment with ABA (A–C) or MeJA (D–F), Figure S3: Venn diagram identifying the unique and shared genes among *PV-NES* and *PV-NLS* transgenic lines after treatment with ABA or MeJA, Figure S4: Interactions of several [Ca^2+^]_cyt_-regulated ABA-responsive genes with four hub genes in tobacco leaves, as determined by LCI assays, Figure S5: Interactions of two [Ca^2+^]_cyt_-regulated JA-responsive genes with three hub genes in tobacco leaves, as determined by LCI assays, Figure S6: Interactions of two [Ca^2+^]_cyt_- and [Ca^2+^]_nuc_-regulated ABA-responsive genes with three hub genes in tobacco leaves, as determined by LCI assays, Figure S7: Interactions of two [Ca^2+^]_cyt_- and [Ca^2+^]_nuc_-regulated JA-responsive genes with three hub genes in tobacco leaves, as determined by LCI assays, Table S1: Primers used in this study, Table S2: [Ca^2+^]-regulated ABA-responsive genes, Table S3: [Ca^2+^]-regulated JA-responsive genes, Table S4: Microarray datasets used for the construction of co-expression networks, Table S5: Decoding Ca^2+^ signalling proteins, Table S6: Hormone signalling pathway proteins, Table S7: Genes in the co-expression network, Table S8: Genes co-expressed with *CAM1*, *CIPK8*, *GAD1*, and *GUN5*.
