# Supplementary material for: Cytosolic and Nucleosolic Calcium-Regulated Molecular Networks in Response to Long-Term Treatment with Abscisic Acid and Methyl Jasmonate in Arabidopsis thaliana
Source: Genes (Basel). 2022 Mar 16;13(3):524. doi: 10.3390/genes13030524 (PMC8950999; doi:10.3390/genes13030524)
Supplement: Supplementary file 1 [file genes-13-00524-s001.zip › Supplementary information.pdf]

Supplementary material

Table S1. Primers used in this study.

Table S2. [Ca^{2+}]-regulated ABA-responsive genes.

Table S3. [Ca^{2+}]-regulated JA-responsive genes.

Table S4. Microarray datasets used for the construction of co-expression networks.

Table S5. Decoding Ca^{2+} signalling proteins.

Table S6. Hormone signalling pathway proteins.

Table S7. Genes in the co-expression network.

Table S8. Genes co-expressed with *CAM1*, *CIPK8*, *GAD1*, and *GUN5*.

Figure S1. Characterisation of *A*. *thaliana* T-DNA insertion mutants *cam1*, *cipk8*, and *gad1*. (A) Schematic diagram for T-DNA insertion in the *A*. *thaliana* genome of *cam1* (SALK_202076c), *cipk8* (SALK_139697c), and *gad1* (SALK_022227). Open box, UTR; black box, exon; line, intron. Bar = 100 bp. (B) Genotypes of *cam1*, *cipk8*, and *gad1* were identified by PCR. *A*. *thaliana* wild-type (WT) was used as the control. *Actin2* was used as the experimental control. LP, LB, and RP primers were used for PCR and are listed in Table S1. (C) Expression patterns of *CAM1*, *CIPK1*, and *GAD1* were detected by RT-PCR in *cam1*, *cipk8*, and *gad1* plants, respectively. WT was used as the control. *Actin2* was used as the experimental control. Primers used for PCR are listed in Table S1.

Figure S2. Volcano plots of the total gene expression profiles of WT, *PV-NES*, and *PV-NLS* transgenic lines after treatment with ABA (A–C) or MeJA (D–F). Each dot represents the mean expression level of individual genes obtained from a normalised microarray dataset. Cut-off

values (yellow lines) were established using the following parameters: fold = |2.0| and P-value with false discovery rate correction = 0.05. Genes above the cut-off values were regarded as differentially expressed genes, either downregulated (green dots) or upregulated (red dots).

Figure S3. Venn diagram identifying the unique and shared genes among *PV-NES* and *PV-NLS* transgenic lines after treatment with ABA or MeJA.

Figure S4. Interactions of several $[Ca^{2+}]_{cyt}$-regulated ABA-responsive genes with four hub genes in tobacco leaves, as determined by LCI assays. Tobacco leaves were transformed with constructs encoding full-length *ndhA*, *psb1*, *PGSIP2*, *CAPE2*, and *AT1G64330* fused with c-LUC, and constructs encoding full-length *GAD1*, *CAM1*, *CIPK8*, and *At1G28400* fused with nLUC. Empty vectors were used as negative controls. Luminance intensity indicates an interaction between any two proteins. Experiments were repeated three times and a representative read-out is shown.

Figure S5. Interactions of two $[Ca^{2+}]_{cyt}$-regulated JA-responsive genes with three hub genes in tobacco leaves, as determined by LCI assays. Tobacco leaves were transformed with constructs encoding full-length *AT3G45160* and *HIR2* fused with cLUC, and constructs encoding full-length *GAD1*, *CAM1*, and *CIPK8* fused with nLUC. Empty vectors were used as negative controls. Luminance intensity indicates an interaction between any two proteins. Experiments were repeated three times and a representative read-out is shown.

Figure S6. Interactions of two $[Ca^{2+}]_{cyt}$- and $[Ca^{2+}]_{nuc}$-regulated ABA-responsive genes with three hub genes in tobacco leaves, as determined by LCI assays. Tobacco leaves were transformed with constructs encoding full-length *RCI2B* and *NTMC2T6.1* fused with cLUC, and constructs encoding full-length *GAD1*, *CAM1*, and *CIPK8* fused with nLUC. Empty vectors were

used as negative controls. Luminance intensity indicates an interaction between any two proteins. Experiments were repeated three times and a representative read-out is shown.

Figure S7. Interactions of two $[Ca^{2+}]_{cyt}$- and $[Ca^{2+}]_{nuc}$-regulated JA-responsive genes with three hub genes in tobacco leaves, as determined by LCI assays. Tobacco leaves were transformed with constructs encoding full-length *AT1G54410* and *AT3G19370* fused with cLUC, and constructs encoding full-length *GAD1*, *CAM1*, and *CIPK8* fused with nLUC. Empty vectors were used as negative controls. Luminance intensity indicates an interaction between any two proteins. Experiments were repeated three times and a representative read-out is shown.

Figure S1

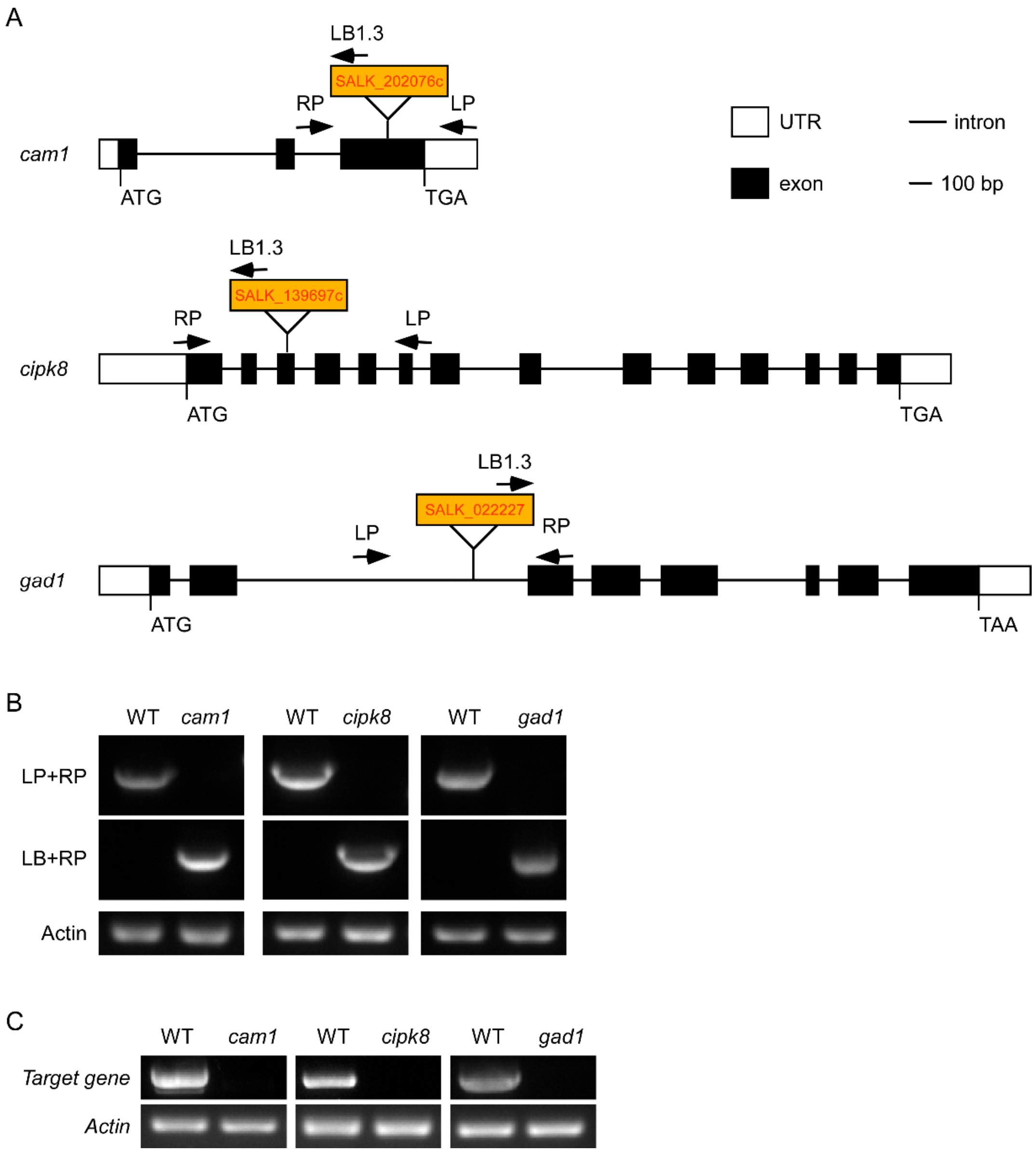

A
LB1.3
SALK_202076c
RP
LP
cam1
ATG
TGA
UTR
intron
exon
100 bp
LB1.3
SALK_139697c
RP
LP
cipk8
ATG
TGA
LB1.3
SALK_022227
LP
RP
gad1
ATG
TAA
B
WT cam1 WT cipk8 WT gad1
LP+RP
LB+RP
Actin
C
WT cam1 WT cipk8 WT gad1
Target gene
Actin

Figure S2

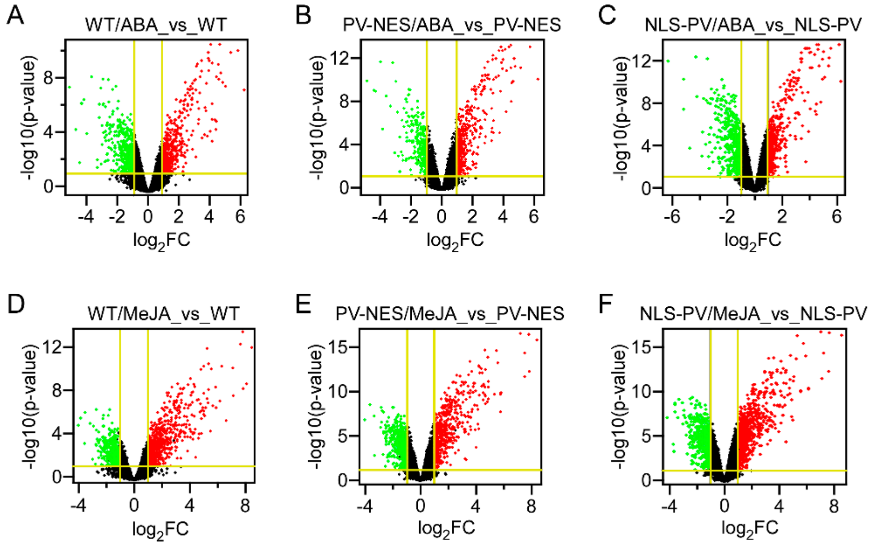
A
WT/ABA_vs_WT
B
PV-NES/ABA_vs_PV-NES
C
NLS-PV/ABA_vs_NLS-PV
D
WT/MeJA_vs_WT
E
PV-NES/MeJA_vs_PV-NES
F
NLS-PV/MeJA_vs_NLS-PV
-log10(p-value)
log2FC

Figure S3

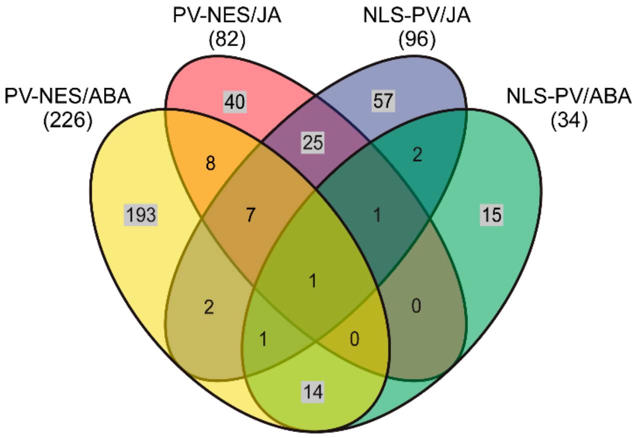
PV-NES/JA
(82)
NLS-PV/JA
(96)
PV-NES/ABA
(226)
NLS-PV/ABA
(34)
40
57
25
8
2
193
7
1
15
1
2
0
1
0
14

Figure S4

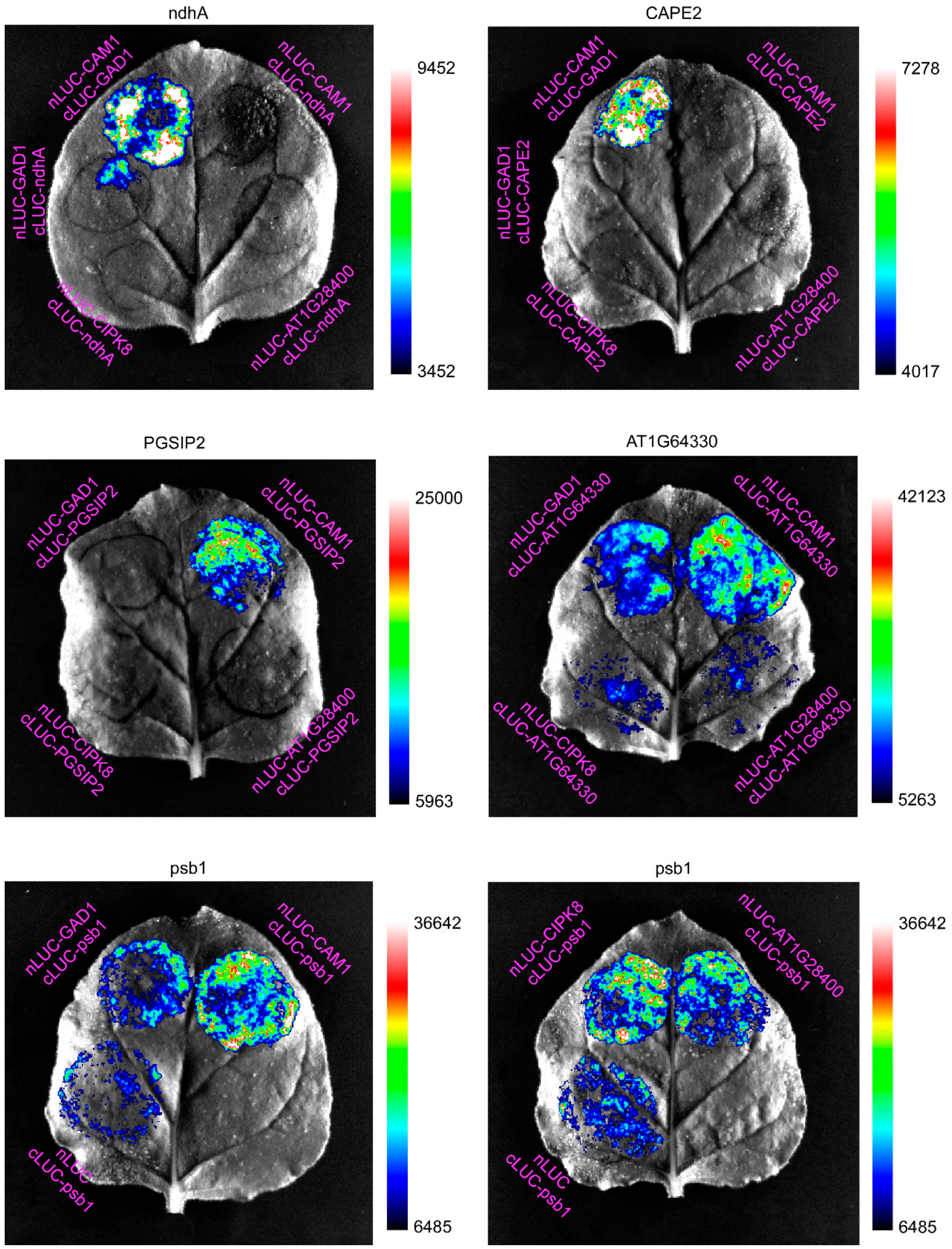

ndhA
nLUC-CAM1
cLUC-GAD1
nLUC-CAM1
cLUC-ndhA
nLUC-GAD1
cLUC-ndhA
nLUC-CIPK8
cLUC-ndhA
nLUC-AT1G28400
cLUC-ndhA
9452
3452
CAPE2
nLUC-CAM1
cLUC-GAD1
nLUC-CAM1
cLUC-CAPE2
nLUC-GAD1
cLUC-CAPE2
nLUC-CIPK8
cLUC-CAPE2
nLUC-AT1G28400
cLUC-CAPE2
7278
4017
PGSIP2
nLUC-GAD1
cLUC-PGSIP2
nLUC-CAM1
cLUC-PGSIP2
nLUC-CIPK8
cLUC-PGSIP2
nLUC-AT1G28400
cLUC-PGSIP2
25000
5963
AT1G64330
nLUC-GAD1
cLUC-AT1G64330
nLUC-CAM1
cLUC-AT1G64330
nLUC-CIPK8
cLUC-AT1G64330
nLUC-AT1G28400
cLUC-AT1G64330
42123
5263
psb1
nLUC-GAD1
cLUC-psb1
nLUC-CAM1
cLUC-psb1
nLUC
cLUC-psb1
36642
6485
psb1
nLUC-CIPK8
cLUC-psb1
nLUC-AT1G28400
cLUC-psb1
nLUC
cLUC-psb1
36642
6485

Figure S5

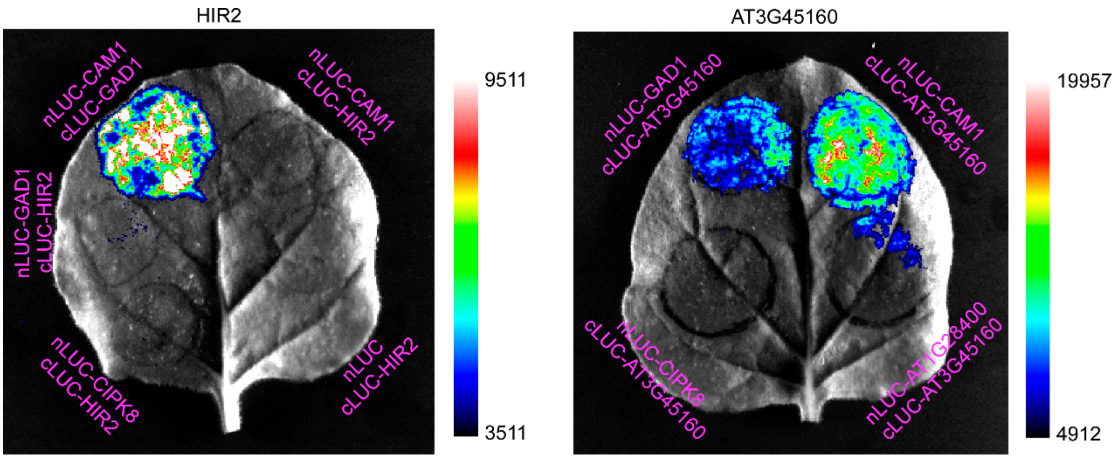
HIR2
nLUC-CAM1
cLUC-GAD1
nLUC-CAM1
cLUC-HIR2
nLUC-GAD1
cLUC-HIR2
nLUC-CIPK8
cLUC-HIR2
nLUC
cLUC-HIR2
9511
3511
AT3G45160
nLUC-GAD1
cLUC-AT3G45160
nLUC-CAM1
cLUC-AT3G45160
nLUC-CIPK8
cLUC-AT3G45160
nLUC-AT1G28400
cLUC-AT3G45160
19957
4912

Figure S6

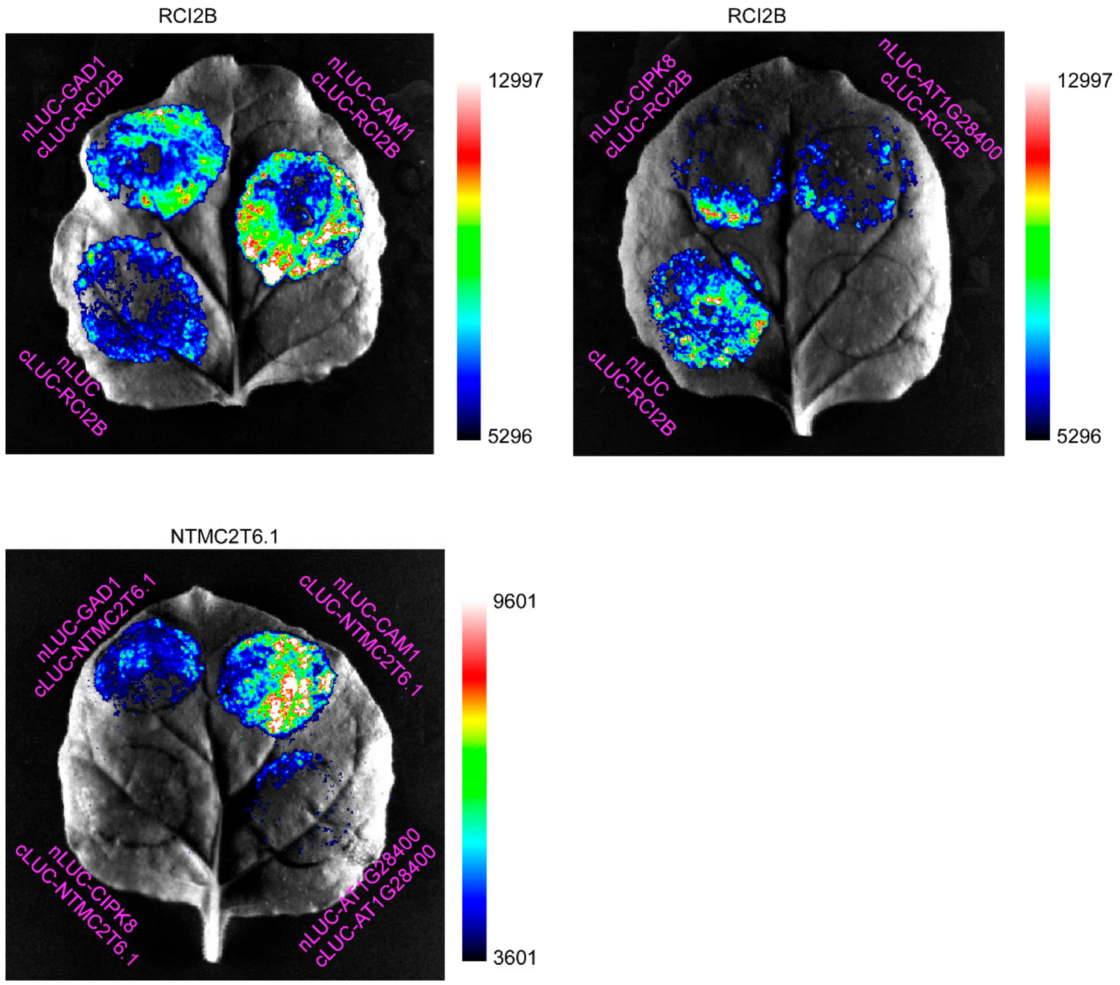
RCI2B
nLUC-GAD1
cLUC-RCI2B
nLUC-CAM1
cLUC-RCI2B
nLUC
cLUC-RCI2B
12997
5296
RCI2B
nLUC-CIPK8
cLUC-RCI2B
nLUC-AT1G28400
cLUC-RCI2B
nLUC
cLUC-RCI2B
12997
5296
NTMC2T6.1
nLUC-GAD1
cLUC-NTMC2T6.1
nLUC-CAM1
cLUC-NTMC2T6.1
nLUC-CIPK8
cLUC-NTMC2T6.1
nLUC-AT1G28400
cLUC-AT1G28400
9601
3601

Figure S7

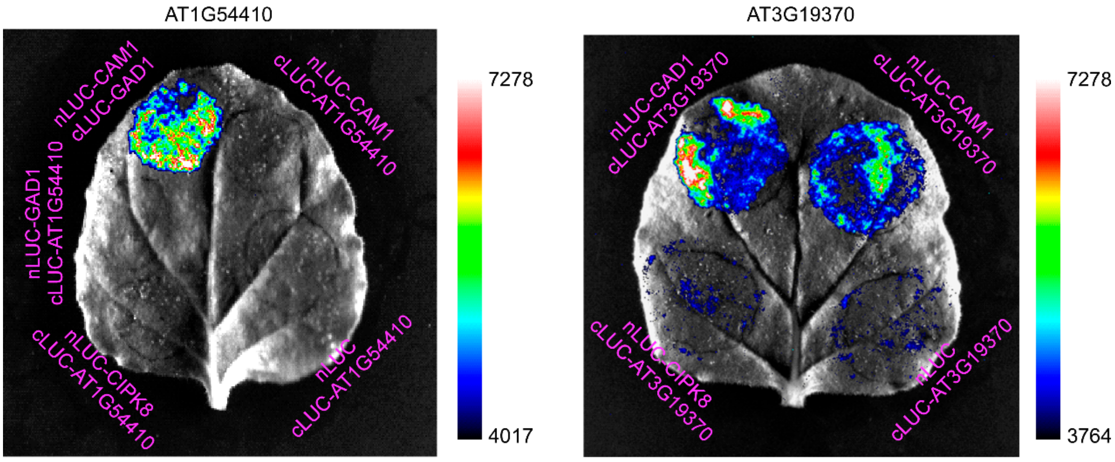
AT1G54410
nLUC-CAM1
cLUC-GAD1
nLUC-CAM1
cLUC-AT1G54410
nLUC-GAD1
cLUC-AT1G54410
nLUC-CIPK8
cLUC-AT1G54410
nLUC
cLUC-AT1G54410
7278
4017
AT3G19370
nLUC-GAD1
cLUC-AT3G19370
nLUC-CAM1
cLUC-AT3G19370
nLUC-CIPK8
cLUC-AT3G19370
nLUC
cLUC-AT3G19370
7278
3764
