# Supplementary material for: Cytosolic and Nucleosolic Calcium-Regulated Molecular Networks in Response to Long-Term Treatment with Abscisic Acid and Methyl Jasmonate in Arabidopsis thaliana
Source: Genes (Basel). 2022 Mar 16;13(3):524. doi: 10.3390/genes13030524 (PMC8950999; doi:10.3390/genes13030524)
Supplement: Supplementary file 1 [file genes-13-00524-s001.zip › Table S1 Primers used in this study.pdf]

**Table S1.** Primers used in this study.

| | Primer | Sequence (5'-3') |
|---|---|---|
| | LB1.3 | ATTTTGCCGATTTCGGAAC |
| Primers for verifying mutant | *cam1* (SALK_202076c) | LP: TCAGCGACCTCTGTTTGTCG<br>RP: GCTTTTACCCATGACAAAACTGC |
| | *cipk8* (SALK_139697c) | LP: AGATGTCACTGAGAACCCCCA<br>RP: CCCCCAACCCCTTGTTACTTT |
| | *gad1* (SALK_022227c) | LP: TTGTTTTTGGGGGAGGTTGGA<br>RP: TTGAACATTGGCTCCGGTGA |
| Primers for detecting RNA level in mutant | *CAM1-F* | GCGATGTGTTTGTTCTCTCTG |
| | *CAM1-R* | CCCATCGGTTTCAATCCAACTTA |
| | *CIPK8-F* | CTTGCCCCTCATATTTTGCC |
| | *CIPK8-R* | CATCGCATCAGACCGTATTC |
| | *GAD1-F* | TTTGTGACGACATGGATGGA |
| | *GAD1-R* | TCAAGGAGAAAGCGACAAGT |
| Primers for qRT-PCR | *ERF104-F* | GACGAAGACCGTGGGGAAAA |
| | *ERF104-R* | AAGCTCTTCCAGCTTCCACG |
| | *NCED3-F* | AGGTCGCAAGATTCGGGATT |
| | *NCED3-R* | GCGGATTTCAGACAGGACACTC |
| | *PR1-F* | CTCATACACTCTGGTGGG |
| | *PR1-R* | TTGGCACATCCGAGTC |
| | *AGL21-F* | CTTCATGCTGGAGCTTGCAAAGTC |
| | *AGL21-R* | AGCTATTCTCTGTGATGCCGAGGT |
| | *CAM1-F* | GATGATGATGACCTTTTTATGC |
| | *CAM1-R* | TTACACACAAAAGTCACAAACC |
| | *CIPK8-F* | AAGGTTCATCTCACACAAGC |
| | *CIPK8-R* | TATTCTTCTGCGTCTCCTGC |
| | *GAD1-F* | GCACCGTTAGAAGAGGCGGA |
| | *GAD1-R* | TTGCCTTCAGCTTTGCGCTT |

| | |
|---|---|
| *Actin2-F* | TAACAGGGAGAAGATGACTCAGATCA |
| *Actin2-R* | AAGATCAAGACGAAGGATAGCATGAG |
