# Supplementary material for: Cytosolic and Nucleosolic Calcium-Regulated Molecular Networks in Response to Long-Term Treatment with Abscisic Acid and Methyl Jasmonate in Arabidopsis thaliana
Source: Genes (Basel). 2022 Mar 16;13(3):524. doi: 10.3390/genes13030524 (PMC8950999; doi:10.3390/genes13030524)
Supplement: Supplementary file 1 [file genes-13-00524-s001.zip › Table S4 Microarray datasets used for the construction of co-expression networks.pdf]

Table S4. Microarray datasets used for the construction of co-expression networks.

GEO no	Arrays	Used	Chip	Cel	Description	Refs
GSE12715	12	7	GPL198	Y	Samples from wild type (Col), aba2 (or gin1-3), etr1-1, and ein2-1, which were grown on 1% sucrose agar plates for 12 to 14 days.	
GSE45662	10	10	GPL198	Y	21-day-old seedlings treated with 100 μM MeJA for various time periods (0, 1, and 4 hours).	(Hu et al., 2013)
GSE84446	48	27	GPL198	Y	8 timepoints after control or ACC treatment done in triplicate.	
GSE39384	72	57	GPL198	Y	Arabidopsis seedlings of the wild-type or hormone mutants were treated with plant hormones.	(Goda et al., 2008)
GSE7432	16	15	GPL198	Y	Ethylene and auxin on the root of mutant or WT Arabidopsis.	(Stepanova et al., 2007)
GSE5620	36	29	GPL198	Y	36 samples used as control in stress treatment.	(Kilian et al., 2007)
GSE109611	29	20	GPL198	Y	3-day-old WT, NES-PV and NLS-PV seedlings treated with 10 μM ABA, 50 μM MeJA and 100 μM ACC for 5 days.	

Goda H, Sasaki E, Akiyama K*, et al.* (2008). The AtGenExpress hormone and chemical treatment data set: experimental design, data evaluation, model data analysis and data access. *The Plant Journal* **55**: 526-542.

Hu P, Zhou W, Cheng Z*, et al.* (2013). JAV1 controls jasmonate-regulated plant defense. *Molecular Cell* **50**: 504-515.

Kilian J, Whitehead D, Horak J*, et al.* (2007). The AtGenExpress global stress expression data set: protocols, evaluation and model data analysis of UV-B light, drought and cold stress responses. *The Plant Journal* **50**: 347-363.

Stepanova A N, Yun J, Likhacheva A V*, et al.* (2007). Multilevel interactions between ethylene and auxin in Arabidopsis roots. *The Plant Cell* **19**: 2169-2185.
